# Neuropathogenesis of acute HIV: mechanisms, biomarkers, and therapeutic approaches

**DOI:** 10.1097/COH.0000000000000923

**Published:** 2025-03-26

**Authors:** Fangzhi (Frank) Jia, Bruce J. Brew

**Affiliations:** a School of Clinical Medicine, Faculty of Medicine and Health, UNSW Sydney RingGold 7800; b Department of Neurology, St Vincent's Hospital, Darlinghurst; c Department of Neurology, Royal North Shore Hospital, St Leonards; d Departments of Neurology and Immunology, Peter Duncan Neuroscience Unit, St Vincent's Hospital, University of New South Wales and University of Notre Dame, Darlinghurst, Sydney NSW, Australia

**Keywords:** antiretroviral therapy, biomarkers, HIV, HIV-associated neurocognitive disorders, neuropathogenesis

## Abstract

**Purpose of review:**

The neuropathogenesis of acute HIV leads to rapid central nervous system (CNS) involvement, characterized by early viral entry, immune activation, and the formation of viral reservoirs. Despite effective antiretroviral therapy (ART), these reservoirs persist, drive neuroinflammation and injury and lead to HIV-associated neurodegenerative disorders (HAND). This review provides an updated synthesis of the mechanisms in acute HIV neuropathogenesis, biomarkers of CNS injury and emerging therapeutic approaches. A deeper understanding of these mechanisms is critical for addressing persistent HAND in ART-treated individuals.

**Recent findings:**

Growing evidence now supports the principal role of infected CD4^+^ T cells in mediating HIV neuroinvasion alongside monocytes, resulting in seeding in perivascular macrophages, pericytes, and adjacent microglia and astrocytes. These reservoirs contribute to ongoing transcriptional activity and viral persistence despite antiretroviral therapy. Neuroinflammation, driven by activated microglia, astrocytes, inflammasomes, and neurotoxic viral proteins, disrupts neuronal homeostasis. Emerging therapies, including latency-reversing agents and transcription inhibitors, show promise in reducing neuroinflammation and reservoir activity.

**Summary:**

Understanding the mechanisms of HIV neuropathogenesis and reservoir persistence has significant implications for developing targeted therapies to mitigate HAND. Strategies to eliminate CNS reservoirs and reduce neuroinflammation should be prioritized to improve long-term cognitive outcomes in people with HIV.

## INTRODUCTION

The central nervous system is a key target of HIV, with neurological complications such as HIV-associated neurocognitive disorders (HAND) remaining prevalent even in the antiretroviral therapy (ART) era, affecting approximately 20–50% of people with HIV (PWH) [1–5]. HAND are classified into three categories: asymptomatic neurocognitive impairment (ANI), characterized by subtle deficits in ≥2 cognitive domains without functional decline; mild neurocognitive disorder (MND), where cognitive impairment interferes with daily activities; and HIV-associated dementia (HAD), a severe form marked by profound memory loss, psychomotor slowing, and behavioural changes (e.g., apathy, irritability). Understanding the neuropathogenesis of HIV is crucial for developing effective therapeutic strategies to mitigate these disorders.

Acute HIV infection involves early central nerve system (CNS) invasion, often occurring within days to weeks following initial systemic infection. CNS invasion occurs rapidly after systemic HIV, facilitated by infected CD4^+^ T cells and monocytes that traverse the blood–brain barrier (BBB) [6–11]. These cells seed viral reservoirs in perivascular macrophages, microglia, and astrocytes, which sustain low-level viral transcription and chronic neuroinflammation despite antiretroviral therapy (ART) [12–18]. Microglia, the resident immune cells of the CNS, become activated by viral proteins such as Tat and gp120, triggering cytokine release and neuronal injury [19]. Similarly, astrocytes contribute to neurotoxicity through dysregulated glutamate homeostasis and inflammatory signalling [20–23]. The interplay between viral persistence, immune activation, and glial dysfunction drives the progression of HAND, as detailed in subsequent sections.

The inability of ART to act directly at the transcription stage of the HIV life cycle and fully penetrate the CNS contributes to ongoing low-level viral replication (both whole virus and viral proteins), persistent inflammation, and continued development of HAND [11,24–27]. The limited CNS penetration of ART highlights the need for adjunctive therapies that target viral reservoirs and reduce neuroinflammation [28,29].

Research is increasingly focusing on therapeutic strategies such as latency-reversing agents, anti-inflammatory therapies, and neuroprotective interventions to address these issues [30,31]. This review will discuss the mechanisms by which HIV enters the CNS, the establishment of viral reservoirs, subsequent neuroinflammatory responses, and emerging therapeutic approaches aimed at improving neurocognitive outcomes. 

**Box 1:**
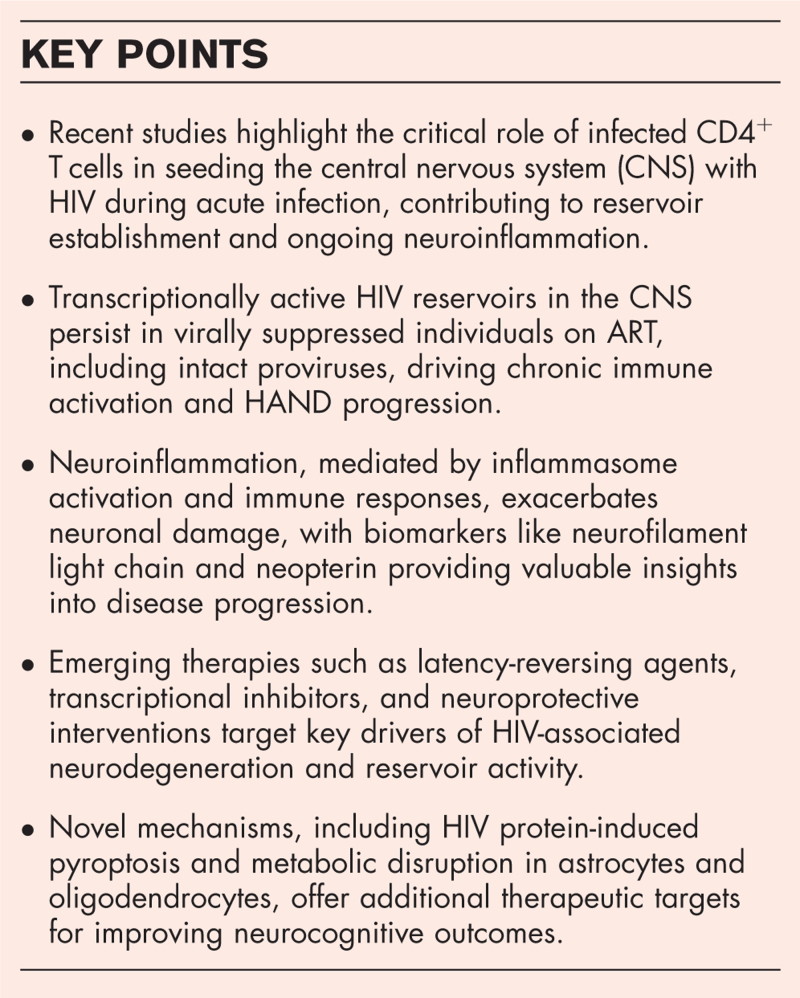
no caption available

### Entry of HIV into the central nervous system

The entry of HIV into the CNS is a crucial early event in the pathogenesis of HAND [16,32]. The process is thought to occur within days to weeks after initial systemic infection, leading to the establishment of viral reservoirs in the CNS, which complicate treatment and facilitate ongoing neuroinflammation [8,9,33^▪^].

HIV enters the CNS through multiple pathways. Foremost is the well accepted “Trojan horse” mechanism, which traditionally depicts infected peripheral monocytes transmigrating across the BBB, differentiating into macrophages, and subsequently establishing viral reservoirs within the CNS [6,34]. BBB integrity is compromised in acute HIV infection due to several factors, including proinflammatory cytokines secreted by perivascular HIV-infected monocytes and macrophages [35]. Monocytes and macrophages are classically thought key contributors to viral entry into the CNS. They are possibly among the first cells to cross the BBB in response to infection, trafficking the virus directly into the brain [1,36]. Upon transmigration into the CNS, these infected macrophages become perivascular macrophages, which not only serve as reservoirs for the virus but also release viral proteins and inflammatory cytokines [14,37].

However, strong accumulating evidence now supports the important and likely principal role that infected T cells, especially CD4^+^ memory T cells, play in HIV neuroinvasion, through entry into the brain and probably choroid plexus during early infection [10,11,38–40]. T cells home to the brain at low numbers to survey the brain, seeding HIV infection [40]. After entry, CD4^+^ T cells probably rapidly transmit the viruses that integrate into perivascular macrophages and microglial cells, the main cell types that harbour HIV replication in the brain [41]. The infected CD4^+^ T cells, however, do not persist long in the brain parenchyma and are generally not found in the brain in postmortem studies. On the other hand, CD8^+^ T cells are found. While infected CD4^+^ T cells are transient in the brain parenchyma, CD8^+^ T cells persist as part of ongoing immune surveillance. This dichotomy may reflect differences in cytopathic effects (HIV preferentially depletes CD4^+^ T cells) or compartmentalized immune responses that limit CD4^+^ survival. Notably, CD8^+^ T cells in the CNS exhibit clonal expansion and effector phenotypes, suggesting sustained antiviral activity [39,40]. Interestingly, a recent discovery showed that a unique population of CD8^+^ T cells which express CD4 on its surface, called CD4^dim^CD8^bright^ T cells, may also mediate HIV neuroinvasion [42] though to a lesser extent than memory CD4^+^ T cells [10,11]. The interactions between HIV-infected T cells and endothelial cells lining the BBB can lead to increased permeability and further facilitate viral entry [43].

Viral proteins, such as Tat and gp120, are also crucial in CNS invasion. These proteins disrupt BBB integrity by inducing oxidative stress, cytokine release, and apoptosis of endothelial cells [7,44]. Tat, in particular, has been shown to activate matrix metalloproteinases (MMPs), enzymes that degrade extracellular matrix components and weaken the BBB [45,46]. On the other hand, gp120-mediated interactions with CXCR4 and CCR5 receptors on endothelial cells further contribute to BBB disruption [47,48].

Another pathway of CNS invasion occurs by the cell-free virus, likely because of BBB permeability due to infiltration by infected mononuclear phagocytes and immune activation [32,49,50]. Further suggested mechanisms include entry through the blood–cerebrospinal fluid barrier and infection of the choroid plexus cells [51,52], and entry through the newly discovered glymphatic system, which may serve as a source of HIV^+^ cells that circulate into the CNS from peripheral lymph nodes [53,54].

### Early immune activation and neuroinflammation

The early immune response to HIV entry in the CNS drives neuroinflammation, contributing significantly to HAND [33^▪^]. Immune activation occurs in a multitude of ways following viral entry and a detailed review on this topic is written by Dr Georgia Tomaras in this issue [55].

Microglia, as resident immune cells, are rapidly activated and express MHC class I and II antigens and adhesion molecules, releasing proinflammatory cytokines like tumour necrosis factor alpha (TNF-α), interleukin (IL)-1β, and IL-6 [15,18]. HIV proteins Tat and gp120 bind to microglial receptors, prompting further inflammation [40,56]. Microglia and astrocytes also release chemokines (e.g., CCL2) which attract monocytes to the CNS across the BBB [21,57,58].

Inflammasomes like NLRP3 also contribute to early neuroinflammation, with activation by viral proteins (e.g. Vpr) resulting in increased pyroptosis and neuronal damage [59–61]. HIV infection triggers the convergent activation of caspases-1 and -3, leading to GSDME- and NINJ1-mediated neuronal pyroptosis, contributing to HAND [62^▪▪^]. A further pathway, the kynurenine pathway, which is involved in energy production and neurodegeneration through its production of quinolinic acid (QUIN) and nicotinamide adenine dinucleotide (NAD), is activated in HAND and several studies have demonstrated a correlation between CSF QUIN levels and HAND severity [22,63,64]. Additionally, HIV-specific T cells infiltrate the CNS and release cytokines, which while controlling viral replication, also promote neuroinflammation [39,65].

### Impact of astrocytes in early infection

Astrocytes are the most abundant glial cells in the CNS and serve crucial roles in maintaining homeostasis and supporting neuronal survival. In the context of HIV, however, these cells are impaired, contributing substantially to neuroinflammation and neuronal dysfunction [1]. Astrocytes and glial cells – through their contributions to neuroinflammation, impaired glutamate handling, cytokine release, and BBB disruption – play a pivotal role in the progression of HIV-associated neuropathology.

Although direct infection of astrocytes by HIV is uncommon due to their relative lack of CD4 receptors, they can nonetheless be infected through other receptors and are intimately involved in HIV neuropathogenesis. HIV can enter astrocytes via endocytosis, but this process is highly inefficient and most viral particles are degraded, an event metaphorically described as a “kiss of death” [66]. Restricted infection of astrocytes occurs more frequently, with up to 19% of astrocytes in infected brain tissue carrying HIV DNA, especially in later disease stages [67]. These infected astrocytes can secrete factors that negatively impact neuronal function, and their activation can lead to the release of neurotoxic substances, including reactive oxygen species (ROS) and glutamate, that contribute to disease [20,56].

A hallmark of HIV-related neuropathology is the dysregulation of astrocytic glutamate handling. Typically, astrocytes work to clear excess extracellular glutamate to protect neurons from excitotoxicity, a form of cell death resulting from overstimulation [68]. However, HIV-infected astrocytes exhibit impaired glutamate uptake and paradoxically release excess glutamate, a process influenced by interactions with infected macrophages and activated microglia [23,69]. This impaired handling of glutamate creates an environment conducive to excitotoxic neuronal damage [70].

The HIV protein Tat can induce astrocytes to express chemokines such as MCP-1 (a chemoattractant for macrophages) and other proinflammatory molecules, thereby promoting the recruitment of immune cells to the CNS and intensifying the inflammatory cycle [71]. Astrocyte activation also involves the release of cytokines like TNF-α, driven by the interaction of stromal cell-derived factor 1 (SDF-1) with CXCR4 receptors, exacerbating inflammation [72].

Although oligodendrocytes are not directly infected by HIV, they are indirectly affected by the ongoing inflammatory milieu. HIV-induced neuroinflammation impairs oligodendrocyte maturation and function, leading to demyelination and subsequent disruption of neuronal connectivity – factors that play a role in the cognitive deficits characteristic of HAND [73–75].

### Neurotoxic viral proteins and neuronal dysfunction

HAND is partly driven by the direct neurotoxic effects of HIV proteins, including gp120, Tat, Vpr, and Nef, which have been implicated in numerous pathways leading to neuronal injury, apoptosis, and dysfunction (Table 1) [7,76]. The effects of these viral proteins are exerted both directly on neurons and indirectly through interactions with glial cells, triggering neuroinflammation and other deleterious processes.

**Table 1:** Neurotoxic HIV proteins

Protein	Mechanism	Impact
Tat	Mitochondrial dysfunction, NMDA receptor activation	Oxidative stress, excitotoxicity
gp120	CXCR4/CCR5 binding, glutamate release	Synaptodendritic injury
Vpr	NLRP3 inflammasome activation	Pyroptosis, neuroinflammation
Nef	Astrocyte cytokine production (TNF-α, IL-6)	Chronic inflammation

IL, interleukin; TNF, tumour necrosis factor.

The HIV envelope protein gp120 interacts with chemokine receptors CCR5 and CXCR4 on neuronal and glial cells, leading to excitotoxicity and calcium dysregulation [77,78]. The binding of gp120 to these receptors results in excessive intracellular calcium influx, which subsequently activates proapoptotic cascades [79,80]. gp120 also induces the release of glutamate from astrocytes, exacerbating excitotoxic damage in neurons – a hallmark of HIV neuropathogenesis [72]. Recent studies have further elucidated the role of gp120 in disrupting synaptic plasticity and reducing dendritic spine density [81,82].

Tat, another HIV protein, is a potent neurotoxin that disrupts mitochondrial function to promote oxidative stress and neuronal apoptosis [83–85]. Tat has also been found to interfere with the function of the *N*-methyl-d-aspartate (NMDA) receptors, enhancing glutamate excitotoxicity and promoting synaptic damage [86,87].

The HIV-1 accessory protein Vpr also contributes to neurodegeneration through its impact on cellular energy metabolism and apoptosis [88]. Vpr disrupts mitochondrial integrity, causing depolarization and the release of pro-apoptotic factors such as cytochrome c, leading to cell death [89,90]. Vpr can also induce neuronal apoptosis indirectly by stimulating the production of proinflammatory cytokines from microglia and astrocytes [91,92]. Additionally, Vpr can alter the permeability of the BBB, exacerbating the influx of infected immune cells into the CNS [93].

Another HIV protein, Nef, stimulates the production of proinflammatory cytokines from glial cells, and it has been linked to dendritic simplification and decreased synaptic density in neurons [94–96]. Nef-induced activation of astrocytes and microglia results in increased levels of tumour necrosis factor-alpha (TNF-α) and interleukin-6 (IL-6), contributing to neuronal dysfunction and apoptosis [97,98].

Recent findings also emphasize the role of these viral proteins in epigenetic modifications that contribute to neuronal dysfunction. For instance, HIV Tat has been shown to alter histone acetylation in neurons, thereby affecting gene expression related to synaptic function and neuronal survival [99,100]. Furthermore, HIV gp120 has been implicated in the dysregulation of microRNAs, which can lead to changes in neuronal gene expression and exacerbate neurodegenerative processes [101].

### Central nervous system reservoir formation and viral persistence despite ART

As described above, neuroinvasion by HIV and establishment of a CNS reservoir occur early, within days to weeks after systemic infection, through infected CD4^+^ T cells and monocytes, subsequently establishing reservoirs in microglia, perivascular macrophages and astrocytes [38,41,102,103]. The brain is a significant HIV reservoir and sanctuary where viral persistence and transcriptional activity continue despite suppressive ART, and this correlates with the reduced, but still high prevalence of HAND in ART-suppressed PWH [104,105^▪^].

Multiple lines of recent evidence have substantiated the presence and ongoing transcriptional activity of the CNS reservoir, and its pathogenicity in relation to HAND. Our recent studies confirmed intact, replication-competent HIV reservoirs in the CNS (frontal lobe) of ART-suppressed PWH [106,107^▪▪^]. Using the highly sensitive Double-R assay, cell-associated HIV-1 RNA transcripts could be quantified in the vast majority of ART-suppressed PWH and occurred at much higher levels in the CSF cells than in their peripheral blood mononuclear cells [11]. Higher levels of HIV-1 transcripts in CSF cells correlated with greater brain injury in the frontal white matter and posterior cingulate [11]. Single-cell analyses of CSF and blood samples in ART-suppressed PWH revealed that a subset of central memory CD4^+^ T cells in the CSF produced HIV-1 RNA and that HIV-1–infected cells were more frequently found in the CSF than in the blood [108].

Further confirming this is a more recent study which showed persistent HIV reservoir in the frontal cortex of suppressed PWH and demonstrated that active HIV transcription occurs [109^▪▪^]. HIV RNA transcripts were detected in frontal cortex tissue of PWH regardless of suppression with ART, and levels of HIV TAR transcripts were similar between suppressed and nonsuppressed PWH [109^▪▪^]. This echoes Henderson *et al.*'s earlier finding that Tat and Trans-activation response (TAR) RNA are present in the CSF and are biologically active in CSF exosomes [110]. Looking at immune profiling, older ART-suppressed PWH still have noticeable interferon, stress response and energy metabolism gene expression changes which correlate with white matter abnormalities [111].

These results have considerable therapeutic implications which will be discussed in a later section. Several classes of ART exist; however, once viral integration has occurred, the only class of drugs that can inhibit formation of replicating viral particles is protease inhibitors. Even so, protease inhibitors prevent the cleavage of the gag-pol polyprotein and have no effect on the production of early viral proteins such as Tat. This has two consequences; one is that Tat protein levels may indicate the presence and potentially the size of the viral reservoir [110], and second is that it partially contributes to the observation that long-term viral suppression with ART does not appear to be impacting the level of transcriptional initiation in the brain [11,109^▪▪^].

### Biomarkers of neuropathogenesis in acute HIV

The identification and monitoring of biomarkers are essential for understanding the neuropathogenesis of acute HIV infection and for assessing the progression of HAND (Table 2). Biomarkers can provide insights into the early immune response, neuronal injury, inflammation, and changes in the integrity of the BBB, reflecting different aspects of HIV's impact on the CNS [112–114].

**Table 2:** CNS Biomarkers of HIV

Biomarker	Source	Association
Neurofilament light chain (NfL)	Neuronal axons	Axonal injury, HAND progression
Neopterin	Activated macrophages/monocytes/microglia	Neuroinflammation
sCD163	Monocyte activation	Cognitive impairment
GFAP	Astrocytes	Cognitive impairment

CNS, central nervous system.

In the early stages of HIV infection, elevated levels of inflammatory cytokines such as IL-6, TNF-α, and interferon-gamma (IFN-γ) are frequently observed, indicating an acute immune response within the CNS [115]. These cytokines are not only markers of immune activation but also contributors to the disruption of neuronal homeostasis. A recent systematic review found that higher levels of IFN-γ, IL-1α, IL-7, IL-8, sTNFR-II and lower levels of IL-6, as well as higher levels of neopterin, sCD163 and sCD14 were consistently associated with neurocognitive impairment [116].

Another important marker of immune activation is neopterin, a byproduct of activated macrophages, monocytes and microglia. Elevated neopterin levels in cerebrospinal fluid (CSF) have been reported in individuals with acute HIV infection, reflecting early immune activation within the CNS [117–120]. Recent research continues to support the association between neopterin levels and neurocognitive outcomes, underscoring its relevance as a biomarker of neuroinflammation [121,122].

Soluble CD14 (sCD14) and soluble CD163 (sCD163), markers of monocyte and macrophage activation respectively, are also elevated during acute HIV infection, indicating ongoing inflammation and the activation of the myeloid compartment in the CNS [123,124]. Elevated sCD163 levels have been linked to neuronal damage and cognitive impairment, making it a valuable marker for assessing the degree of inflammation-related neurotoxicity [125]. Recent studies also highlight the association of sCD14 and sCD163 levels with persistent cognitive deficits in individuals on ART [126].

Neurofilament light chain (NfL) is a key biomarker for neuronal damage and HAND risk, elevated in acute and chronic HIV infection [122,127,128]. Recent findings suggest that NfL levels remain elevated in some patients despite effective ART, indicating ongoing subclinical neurodegeneration [129].

Markers of oxidative stress, such as F2-isoprostanes, are also elevated during acute HIV infection, suggesting that ROS production and lipid peroxidation are involved in the early neuropathogenetic processes [130,131]. BBB disruption is another critical aspect of acute HIV neuropathogenesis, and markers like MMP-9 and S100β, a calcium-binding protein produced by astrocytes, have been used to assess BBB integrity [64,132,133]. S100β levels are also elevated in the CSF of individuals with acute HIV infection and have been correlated with neuroinflammation and cognitive deficits [134].

Recent advances in proteomic analyses have revealed differential expression of proteins related to immune activation, synaptic function, and mitochondrial dynamics in the CSF and plasma of HIV-infected individuals [135,136]. These high-throughout approaches have identified potential new biomarkers, such as mitochondrial DNA (mtDNA) and extracellular vesicles (EVs), which may provide insights into mitochondrial dysfunction and intercellular communication during acute HIV infection [137]. A recent study identified plasma glial fibrillary acidic protein (GFAP) as a novel biomarker of cognitive decline in PWH [138].

MicroRNAs (miRNAs) are also emerging as important biomarkers for assessing neuropathogenesis in acute HIV infection. Dysregulation of miRNAs, such as miR-9, miR-29, and miR-146a, has been observed in HIV-infected individuals and has been linked to neuroinflammatory and neurodegenerative processes [101,139]. The expression patterns of these miRNAs are associated with the severity of HAND and may serve as noninvasive biomarkers for monitoring disease progression [140].

### Therapeutic considerations in view of HIV neuropathogenesis

Early ART initiation during acute HIV significantly mitigates neuropathogenesis. The Strategic Timing of Antiretroviral Treatment (START) trial found that immediate ART reduces CNS viral reservoirs compared to delayed treatment [33^▪^]. Early suppression limits immune activation, as evidenced by lower CSF neopterin and neurofilament light chain (NfL) levels in rapid initiators [141]. These findings underscore the importance of universal ‘test-and-treat’ strategies to prevent HAND.

Despite the success of ART in transforming HIV into a manageable chronic condition, HAND remains a persistent challenge, driven largely by immune activation, chronic inflammation, and the persistence of viral reservoirs in the CNS. New therapeutic approaches aim to complement ART by addressing these underlying drivers of neurocognitive impairment [30].

One key area of focus has been developing adjunctive therapies that specifically target immune activation and neuroinflammation. Anti-inflammatory agents, such as minocycline and statins, have shown limited benefit in reducing inflammation without consistent effect on cognitive outcomes [142–146]. A 2022 trial showed ruxolitinib, a Janus kinase (JAK) inhibitor, was well tolerated and significantly decreased markers of immune activation and cell survival in ART-treated PWH [147].

Another promising approach involves the use of neuroprotective agents aimed at preventing neuronal damage. Memantine, an NMDA receptor antagonist, and lithium, a mood stabilizer, have been studied for their potential neuroprotective effects in HIV. However, clinical outcomes have been mixed, with limited efficacy reported for memantine [148].

The gut-brain axis contributes to HAND pathogenesis through microbial translocation and neuroinflammation in ART-suppressed HIV infection [149]. Probiotics, prebiotics, and faecal microbiota transplantation (FMT) are being investigated as possible interventions to restore gut integrity and reduce inflammation, potentially benefiting neurocognitive health [150,151].

Addressing the persistence of HIV within the CNS, novel strategies involving latency-reversing/reactivating agents and gene therapy are also under exploration [152,153]. Latency-reversing agents like histone deacetylase (HDAC) inhibitors aim to reactivate latent HIV (“shock and kill”), which could help reduce viral reservoirs in the CNS [154]. Another strategy to incapacitate the ability of HIV reservoir to reactivate is the “block and lock” approach, using the so-called latency-promoting agents, which can inhibit HIV transcription by inducing a deep latency state [155]. These agents are largely experimental, but the principle of transcription inhibition is paramount in future HIV drug research, since it may be argued that there is no true latency state in the brain, i.e. there is continuing transcriptional activity which underlies neurodegeneration. Meanwhile, gene-editing technologies like CRISPR-Cas9 have shown potential for excising proviral DNA, although their clinical application faces significant challenges related to effective delivery and safety [156–158].

Improving the ability of ART to cross the blood-brain barrier (BBB) is another therapeutic focus. The CNS penetration-effectiveness (CPE) score has been used to evaluate ART regimens, with higher CPE scores being linked to better viral suppression in the CNS and improved cognitive outcomes in some but not all studies [26]. The variable results may reflect the heterogeneity of HAND patients with respect to BBB impairment – when it is present the CPE score may not be relevant as the drug can enter regardless. Recent efforts have centred on the development of nanoformulations of antiretrovirals to enhance drug delivery to the CNS, which could be instrumental in reducing viral persistence and inflammation [159].

## CONCLUSION

The acute neuropathogenesis of HIV involves early CNS entry through infected CD4^+^ T cells and monocytes, establishing reservoirs that fuel neuroinflammation. Mechanisms include microglial activation, leading to cytokine release and neuronal damage, and astrocytic dysfunction causing excitotoxicity. Establishment of a CNS reservoir, viral persistence and replication, and central neuroinflammation occur despite ART suppression and drive ongoing neurodegeneration. Biomarkers such as neopterin, TNF-α, IL-1β, and neurofilament light chain (NfL) indicate inflammation and neuronal injury, aiding in monitoring disease progression. Therapeutic strategies focus on reducing neuroinflammation via anti-inflammatory agents, enhancing ART penetration into the CNS, targeting latent reservoirs using latency-reversing agents and inhibiting transcriptional activity. Addressing these processes is crucial to prevent early neuronal injury and mitigate long-term cognitive decline associated with HIV infection.

## Acknowledgements

*None.*

### Financial support and sponsorship

*None.*

### Conflicts of interest

*There are no conflicts of interest.*

## REFERENCES AND RECOMMENDED READING

Papers of particular interest, published within the annual period of review, have been highlighted as:

▪ of special interest
▪▪ of outstanding interest
